# Circular RNA profiling and its potential for esophageal squamous cell cancer diagnosis and prognosis

**DOI:** 10.1186/s12943-018-0936-4

**Published:** 2019-01-23

**Authors:** Liyuan Fan, Qiang Cao, Jia Liu, Junpeng Zhang, Baosheng Li

**Affiliations:** 1 Department of Radiation Oncology, Shandong Cancer Hospital affiliated to Shandong University, Jinan, 250117 China; 2 School of Computer Science and Engineering, Southeast University, Nanjing, China; 3 Department of Clinical Laboratory, the Second Hospital of Shandong University, Jinan, China

**Keywords:** Circular RNAs, Esophageal squamous cell cancer, Biomarkers, hsa_circ_0001946, Exosome

## Abstract

Esophageal squamous cell cancer (ESCC) is a high incidence and mortality disease worldwide. However, specificity and sensitivity of its diagnostic and prognostic biomarkers are still unsatisfactory. Recently, circular RNAs (circRNAs) as biomarkers have been studied extensively while the expression profile and clinical significance of circRNAs in ESCC have rarely been studied. We performed circular RNA microarray in 3 pairs of ESCC frozen tumor and non-tumor tissues to identify ESCC-related circRNAs and found 1045 up-regulated and 1032 down-regulated circRNAs among which 6 circRNAs (hsa_circ_0062459, hsa_circ_0076535, hsa_circ_0072215, hsa_circ_0042261, hsa_circ_0001946, and hsa_circ_0043603) displayed consistency with microarray results by qRT-PCR. 3 circRNAs (hsa_circ_0062459, hsa_circ_0001946, and hsa_circ_0043603) were also detected in plasma and 2 of them except hsa_circ_0062459 could be used as diagnostic biomarkers and found in exosome of cell-conditioned culture conditioned media. The AUC, sensitivity and specificity of hsa_circ_0001946 were 0.894, 92, 80%, of hsa_circ_0043603 were 0.836, 64, 92% while a signature combining them were 0.928, 84 and 98%. Hsa_circ_0001946 was confirmed to predict the recurrence, overall survival (OS) and disease-free survival (DFS) in frozen and FFPE tissues, while its overexpression decreased cell proliferation, migration, and invasion.

**Electronic supplementary material:**

The online version of this article (10.1186/s12943-018-0936-4) contains supplementary material, which is available to authorized users.

## Main text

Esophageal cancer (EC) is a common cancer type with high incidence and mortality rate, which is characterized by the international variance of incidence rate and pathologic patterns [1]. The highest incidence rates are found in Eastern Asia and in Eastern and Southern Africa, while 90% of cases were ESCC in this area [2]. However, its diagnosis and prognostic prediction confronted the following three challenges: the late appearance of symptoms, cost or uncomforting of endoscopy, the insensitivity, and non-specificity of biomarkers [3]. Therefore, new sensitive and cost-effective biomarkers for ESCC diagnosis and prognosis are urgently needed.

CircRNAs are could be free from RNA exonuclease and exhibits higher stability than messenger or linear noncoding (NC) RNA [4]. Additionally, circRNA not only exists intracellular space but also could export into extracellular space [5]. This trait made it possible to utilize blood as biomarkers detecting samples, which is an less invasive method. What’s more, circRNAs were verified to express by specific cell types or in particular pathological conditions [6]. Several circRNAs were confirmed as diagnostic or prognostic biomarkers in cancer, including glioma [7], lung cancer [8], and laryngeal squamous cell carcinomas [9] etc. However, the usage of circRNAs as biomarkers in ESCC is still lack of exploration. Hence, we performed a prospective study (from Sep 2016 to May 2018) to find potential circRNA biomarkers, which was composed of four parts (Additional file 1**: **Figure S1). We demonstrated that circRNAs could be used for ECSS diagnosis or prognostic prediction and as promising targets for ESCC treatment.

## CircRNA expression profile and validation of circRNAs expression in frozen tissues

We conducted high-throughput human circRNA microarray to assess the differences of circRNA expression profiles between ESCC frozen tumor and non-tumor tissues(Additional file 2**:** Figure S2).To verify the results of microarray and identify the most possible clinical biomarkers, we ranked up-regulated and down-regulated DE circRNAs respectively according to fold changes, *P* value, processed signal value and the number of miRNA-responsive elements (MREs). 8 circRNAs were picked (Additional file 3**:** Table S1) and 6 circRNAs (hsa_circ_0062459, hsa_circ_0076535, hsa_circ_0072215, hsa_circ_0042261, hsa_circ_0001946, and hsa_circ_0043603) were consistent with the microarray result by qRT-PCR in another 10 pairs of tissues (Additional file 4**:** Figure S3). Then we expanded sample size to 50 pairs to measure the expression level of these 6 DE circRNAs (Additional file 5**:** Figure S4) and explore their relationship with clinicopathological characteristics of ESCC patients (Additional file 6**:** Table S2).

## Hsa_circ_0001946 and hsa_circ_0043603 as potential diagnostic biomarkers in plasma and secreted by exosomes

Since blood is the most commonly used sample in laboratory medicine and blood test is a less invasive method in clinical medicine, we decided to found circRNAs secreted to blood among these 6 ones mentioned above and assessed their value as diagnostic biomarkers. It was shown that hsa_circ_0042261, hsa_circ_0072215, and hsa_circ_0076535 were neither found in patients’ nor healthy people’s plasma and serum but existed in both frozen tumor and non-tumor tissues (Additional file 7**:** Table S3).

**Fig. 1:**
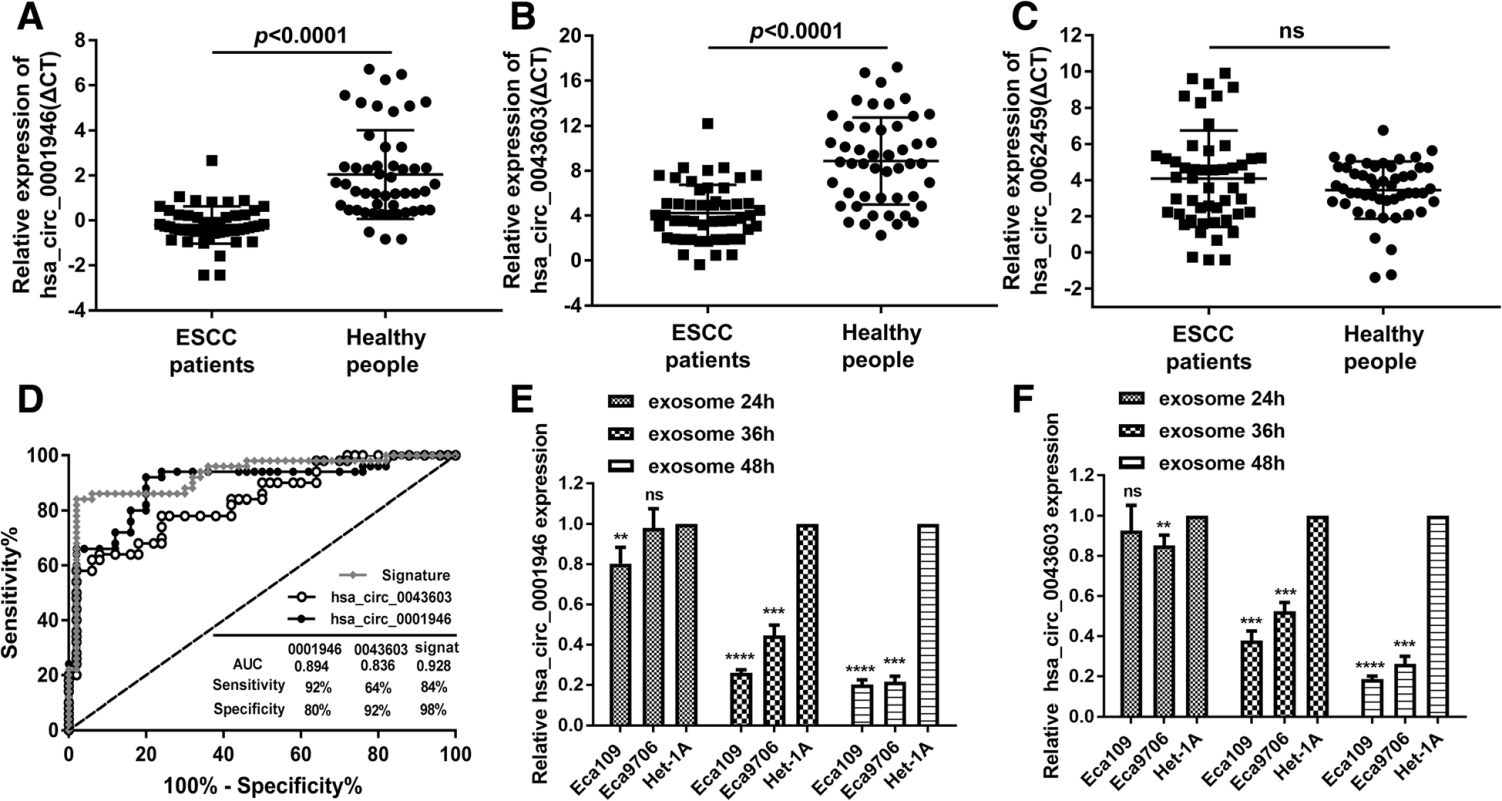
Expression pattern of circRNAs by qRT-PCR in plasma and exosome from cell culture conditioned media. **a**~**c** The expression levels of 3 circRNAs were detected by qRT-PCR in plasma from 50 ESCC patients and 50 healthy people. Student’s t-test was used for differential analysis and data were presented as the means± SEM. **d** Receiver operating characteristic (ROC) curve analysis of differentially expressed circRNAs between ESCC patients and healthy controls. The signature was a combination of hsa_circ_0043603 and hsa_circ_0001946. The AUC, sensitivity, and specificity values were given in the graph. AUC: Area Under The Curve; 0043603: hsa_circ_0043603; 0001946: hsa_circ_0001946; signat:signature. **e**~**f** The expression levels of hsa_circ_0001946 and hsa_circ_0043603 were detected by qRT-PCR in exosome from cell culture conditioned media of Eca109, Eca9706 and Het-1A. One-way ANOVA was used for differential analysis and data were presented as the means± SEM

Then, another 50 pre-operative plasma from patients diagnosed as ESCC by pathological findings were collected while 50 plasma from healthy people were gathered as control. Levels of hsa_circ_0001946, hsa_circ_0043603, and hsa_circ_0062459 were detected in these samples. We found that expression levels of hsa_circ_0001946 (Fig. 1a) and hsa_circ_0062459 (Fig. 1b) in pre-operative plasma were lower than that in healthy people but hsa_circ_0043603 (Fig. 1c) was of no significance. The results indicated that these 2 circRNAs could be used as diagnostic biomarkers. Then the sensitivity and specificity were evaluated by ROC curve analysis. As the results showed, the AUCs of hsa_circ_0001946 and hsa_circ_0062459 were 0.894(sensitivity: 92%, specificity: 80%) and 0.836(sensitivity: 64%, specificity: 92%) respectively. What’s more, we performed a logistic regression to establish circRNAs signature by combining these two circRNAs expression levels in plasma. The formula is 3.272-(0.465*level of hsa_circ_0001946) – (1.706* level of hsa_circ_0062459). The performance was also evaluated by ROC curve analysis. The logistic regression model provided a better diagnostic accuracy, with the AUC, sensitivity, and specificity of 0.928, 84 and 98%, respectively (Fig. 1d). Then the relationship of these 2 circRNAs with clinicopathological characteristics of ESCC was also further studied (Additional file 8**:** Table S4).

Since hsa_circ_0001946 and hsa_circ_0043603 may be potential diagnostic biomarkers, we then explored the origin of circRNAs in plasma. We used cultured cell model and collected the CCM to isolate exosomes. The copy number differences of hsa_circ_0001946 and hsa_circ_0043603 in esophageal epithelial cells and ESCC cells as well as the copy number changes along with time were studied. We found that expression levels of hsa_circ_0001946 and hsa_circ_0043603 in exosomes differed in esophageal epithelial cells and ESCC cells and the difference increased in a time-dependent manner (Fig. 1e and Fig. 1f). This inferred that both esophageal epithelial cells and ESCC cells secreted circRNAs by exosomes, which explained the detectable circRNAs in patients’ plasma to some extent.

## Hsa_circ_0001946 as a prognostic biomarker in both frozen and FFPE tissues

**Fig. 2:**
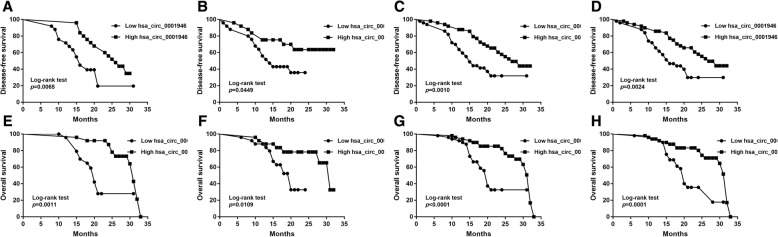
Prognostic significance of hsa_circ_0001946 in ESCC patients. **a** Kaplan-Meier analysis of disease-free survival (DFS) based on hsa_circ_0001946 expression in 50 frozen tumor tissues of ESCC patients by qRT-PCR. **b** Kaplan-Meier analysis of DFS based on hsa_circ_0001946 expression in 50 FFPE tissues of ESCC patients by qRT-PCR. **c** Kaplan-Meier analysis of DFS based on hsa_circ_0001946 expression in 50 frozen tumor tissues and 50 FFPE tissues of ESCC patients by qRT-PCR. **d** Kaplan-Meier analysis of DFS based on hsa_circ_0001946 expression in 100 FFPE tissues of ESCC patients above by FISH. **e** Kaplan-Meier analysis of overall survival (OS) based on hsa_circ_0001946 expression in 50 frozen tumor tissues of ESCC patients by qRT-PCR. **f** Kaplan-Meier analysis of OS based on hsa_circ_0001946 expression in 50 FFPE tissues of ESCC patients by qRT-PCR. **g** Kaplan-Meier analysis of OS based on hsa_circ_0001946 expression in 50 frozen tumor tissues and 50 FFPE tissues of ESCC patients by qRT-PCR. **h** Kaplan-Meier analysis of OS based on hsa_circ_0001946 expression in 100 FFPE tissues of ESCC patients above by FISH

As Additional file 6**:** Table S2 showed, hsa_circ_0001946 was the only one associated with the recurrence rate of ESCC patients. As for disease-free survival (DFS) and overall survival (OS) prediction, patients in the high hsa_circ_0001946 group (according to the median level) had a much shorter DFS and OS (Fig. 2a and Fig. 2e), whose hazard ratio (HR) and 95% confidence interval (CI) were 0.357(0.164–0.781) and 0.209(0.076–0.579) respectively.

Furthermore, we used FFPE tissues to verify this conclusion. The results showed that high hsa_circ_0001946 was associated with shorter DFS and OS in K-M curves (Fig. 2b and Fig. 2f) while univariate Cox proportional hazard models showed that expression of hsa_circ_0001946 in FFPE tissues was an independent prognostic indicator of OS but not DFS. The multivariate Cox proportional hazard models shown that combination of these two group supports the result that hsa_circ_0001946 was a promising and independent prognostic biomarker for ESCC patients in both frozen and FFPE tissues.

And then, we used FISH for semi-quantitation and location of hsa_circ_0001946. As Additional file 9**:** Figure S5d showed, hsa_circ_0001946 locates in the cytoplasm. The above 100 patients’ FFPE tissues were conducted FISH analysis to explore the potential predictive performance for prognosis. The results (Fig. 2d, Fig. 2h and Additional file 10**:** Table S5) showed that expression of hsa_circ_0001946 by FISH was associated with DFS and OS and an independent prognostic factor.

## Hsa_circ_0001946 targeted miRNA-mRNA network and its role in cell proliferation, migration, and invasion

Increasing studies have revealed the sponge role of circRNA to miRNA by a conserved seed sequence. We also conducted miRNA microarray in the same ESCC frozen samples and performed coexpression analysis (Additional file 11**:** Figure S6a). The result was conjoint with predicted miRNAs bond to hsa_circ_0001946 based on miRanda and miRNA-7-5P stood out from the crowd. Then we make target prediction of miRNA-7-5p by starBase and 1597 targeted mRNA were found. We picked 6 genes which were positive in all 5 algorithms (TargetScan, PicTar, RNA22, PITA and miRanda). As it was shown in Additional file 11**:** Figure S6b. Finally, we performed GO and KEGG analysis as well as miRNA cluster analysis by DIANA tool (Additional file 11**:** Figure S6c and Figure S6d).

**Fig. 3:**
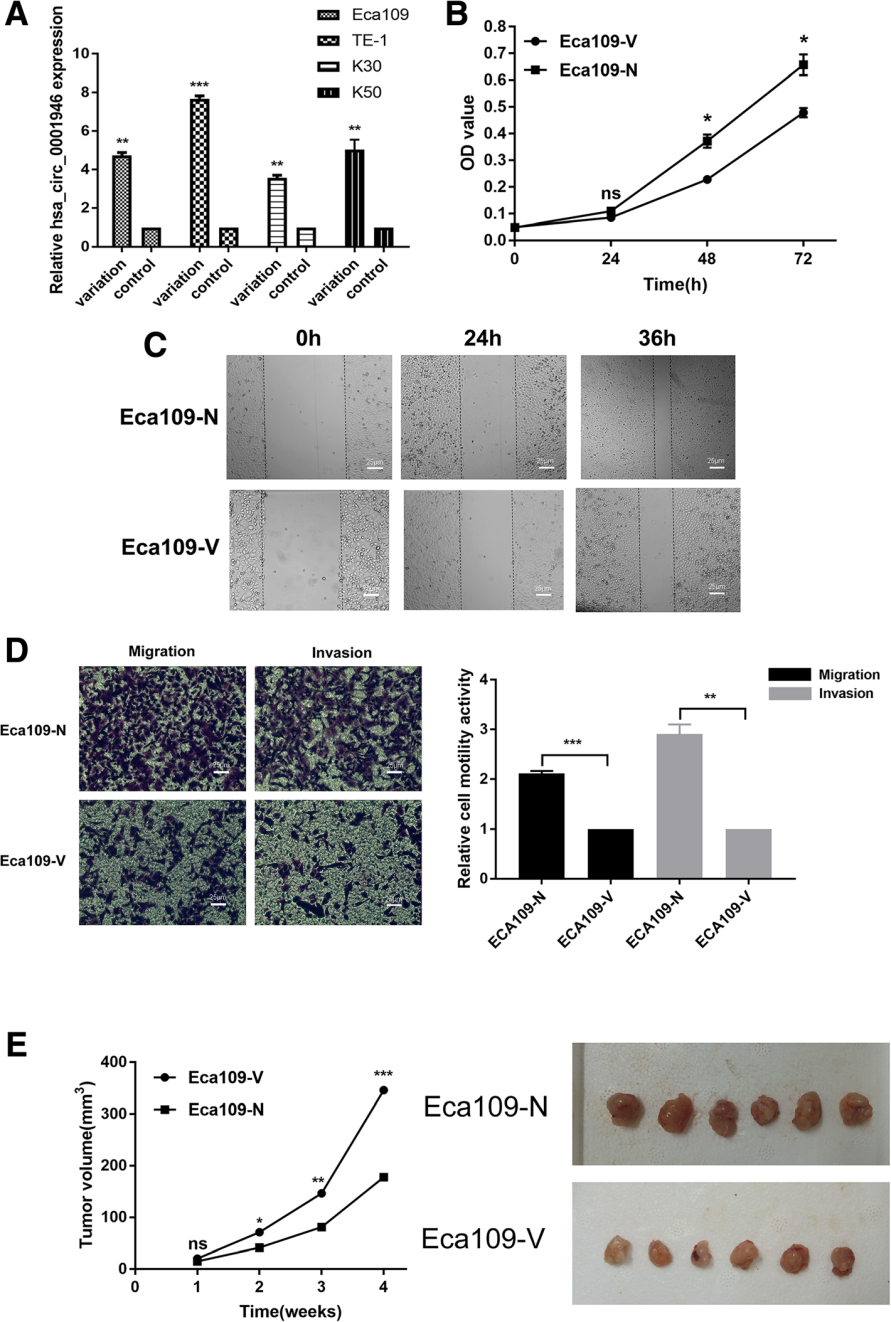
Hsa_circ_0001946 overexpression affects the proliferation, migration, and invasion of Eca109. (**a**) ESCC cell lines (Eca109, TE-1, K30, and K50) were transfected with lentivirus and the expressions of hsa_circ_0001946 were elevated a lot by qRT-PCR. (**b**) MTT method showed that hsa_circ_0001946 overexpression inhibited Eca109 proliferation after 48 h. (**c**) Wound-healing assay indicated that hsa_circ_0001946 overexpression decreased Eca109 migration after 24 h. (**d**) Transwell assays showed that hsa_circ_0001946 overexpression decreased Eca109 migration and invasion after 48 h incubation. (*n* = 3; Student’s t-test was used for significance test and data were presented as the means± SEM). (E) Subcutaneous xenografts excised from nude mice. (*n* = 6;Student’s t-test was used for significance test and data were presented as the means± SEM)

We next explored the role of hsa_circ_0001946 in pathogenic mechanism of ESCC in vitro. Firstly, stable cell line overexpressing hsa_circ_0001946 was built by lentiviral transduction into Eca-109, TE-1, K-30, K-50 and named Eca-109-V, TE-1-V, K-30-V, K-50-V while the control cell lines were named Eca-109-NC, TE-1-NC, K-30-NC, K-50-NC. The lentiviral transduction elevated hsa_circ_0001946 expression a lot in these cell lines (Fig. 3a). MTT assay showed that hsa_circ_0001946 overexpression significantly decreased the proliferation of Eca109 (Fig. 3b). Wound-healing assay exhibited that hsa_circ_0001946 overexpression excessively decreased the migration of Eca109 (Fig. 3c). Transwell assay with or without Matrigel revealed that hsa_circ_0001946 overexpression meaningfully decreased the migration and invasion of these Eca109 (Fig. 3d). Moreover, in vivo experiment showed that hsa_circ_0001946 overexpression significantly decreased the proliferation of Eca109 (Fig. 3e) and TE-1 but not K30(Additional file 12**:** Figure S7e). The results also showed that hsa_circ_0001946 overexpression significantly decreased the proliferation, migration, and invasion of TE-1, K-30, K-50 (Additional file 12**:** Figure S7).

## Conclusion

In conclusion, our study reveals the roles of circRNAs in ESCC. 2 circRNAs had shown the potentials as blood biomarkers for ESCC. Hsa_circ_0001946 exhibited promising potency to be a prognostic biomarker and play roles in oncogenesis development of ESCC. However, based on these findings, further validation and underlying mechanism are still in request.

## Additional files

Additional file 1: **Figure S1.** Flowchart of our study. P1 to P4 represents four parts. DE: differentially expressed; PCR: Polymerase Chain Reaction; FISH: Fluorescence In Situ Hybridization; FFPE: formalin fixed paraffin-embedded; GO: Gene Ontology; KEGG: Kyoto Encyclopedia of Genes and Genomes. (PDF 2574 kb)

Additional file 2: **Figure S2.** Screening of differentially expressed circRNAs by circRNA microarray and functional annotation of their host genes. (A) Hierarchical clustering results of circRNAs expression profiles among 3 pairs of ESCC tumor tissues and non-tumor tissues. Red represented relatively high expression while green represented relatively low expression. A1~C1 were tumor tissues while A2~C2 were non-tumor tissues. (B) Red dots in the scatter-Plot indicated high expressed circRNAs while green dots here indicated low expressed circRNAs. (C) Go analysis of host genes was performed to obtain three categories (cellular component, molecular function, and biological process). (D) The top 30 signaling pathways potentially involved in the circRNA-mediated regulatory network in ESCC by KEGG analysis of host genes. (PDF 3924 kb)

Additional file 3: **Table S1.** Selected DE circRNAs by microarray and validation by qRT-PCR. *number of MRE targeted by the related circRNA; DE: differentially expressed; FC: fold change; MRE: miRNA response elements (DOCX 17 kb)

Additional file 4: **Figure S3.** Confirmation of differentially expressed circRNAs by qRT-PCR in frozen tumor and non-tumor tissues. (A)~(H)The expression levels of 8 circRNAs(hsa_circ_0062459, hsa_circ_0076535, hsa_circ_0072215, hsa_circ_0033872, hsa_circ_0042261, hsa_circ_0070809, hsa_circ_0001946 and hsa_circ_0043603) were detected by qRT-PCR in 10 pairs of frozen tissues. Mann-Whitney test was used for the significance test. (PDF 1728 kb)

Additional file 5: **Figure S4.** Confirmation of differentially expressed circRNAs by qRT-PCR in frozen tumor and non-tumor tissues. (A)~(F) The expression levels of 6 circRNAs(hsa_circ_0062459, hsa_circ_0076535, hsa_circ_0072215, hsa_circ_0042261, hsa_circ_0001946, and hsa_circ_0043603) were detected by qRT-PCR in 50 pairs of frozen tissues. Student’s t-test was used for significance test. (PDF 2114 kb)

Additional file 6: **Table S2.** Relationships of circRNAs expression levels in ESCC frozen tumor tissues with clinicopathological characteristics by qRT-PCR. (DOCX 21 kb)

Additional file 7: **Table S3.** Average expression of 6 circRNAs in 6 ESCC patients’ samples and 6 healthy people’s samples. *It means hsa_circ_0042261 was detected in one samples among total 6 samples (DOCX 14 kb)

Additional file 8: **Table S4.** Relationships of circRNAs expression levels in plasma of ESCC patients with clinicopathological characteristics by qRT-PCR. (DOCX 18 kb)

Additional file 9: **Figure S5.** Semi-quantitation and location of hsa_circ_0001946 by FISH in FFPE tissues. (A~B) High and low expression levels of hsa_circ_0001946 in samples by Immunofluorescence Accumulation Optical Density (IOD) analysis under 200X condition. (C) Expression pattern of hsa_circ_0001946 under 400X condition. (D) This image was recorded on a wide-field fluorescence microscope via a 63× oil objective. It represented the localization of hsa_circ_0001946 in cells. The blue color was stained nuclei by DAPI, and the green color was stained hsa_circ_0001946 in the cytoplasm. (PDF 32764 kb)

Additional file 10: **Table S5.** Univariate and multivariate Cox regression analyze hsa_circ_0001946 for overall survival (OS) and disease-free survival (DFS) of patients in frozen tumor tissue, FFPE tissue and plasma of ESCC patients. (DOCX 17 kb)

Additional file 11: **Figure S6.** Prediction an annotation of hsa_circ_0001946 targeted miRNA-mRNA network. (A) The coexpression network of circRNAs and miRNAs obtained by microarray tested in the same three pairs of ESCC tumor tissues and non-tumor tissues. Green ones represent miRNAs while yellow ones represent circRNAs. (B) Hsa_circ_0001946 targeted miRNA-mRNA network combined with the microarray results and predicted by several algorithms. Yellow one represents algorithms hsa_circ_0001946. Pink and green ones represent targeted miRNAs of hsa_circ_0001946. Blue ones represent targeted mRNAs of hsa-miR-7-5P. (C~D) The GO and KEGG analysis of the targeted miRNAs of hsa_circ_0001946. (E~F) The miRNA cluster analysis of the targeted miRNAs of hsa_circ_0001946. (PDF 9920 kb)

Additional file 12: **Figure S7.** Hsa_circ_0001946 overexpression affects the proliferation, migration, and invasion of K30, K50, and TE-1. (A)(D)(G) MTT method showed that hsa_circ_0001946 overexpression inhibited K30, K50, and TE-1 proliferation after 24 h. (B)(E)(H) Wound-healing assay indicated that hsa_circ_0001946 overexpression decreased K30, K50 and TE-1 migration after 24 h. (C)(F)(I) Transwell assays showed that hsa_circ_0001946 overexpression decreased K30, K50 and TE-1 migration and invasion after 48 h incubation. (*n* = 3; Student’s t-test was used for significance test and data were presented as the means± SEM.) (J)Subcutaneous xenografts excised from nude mice. (*n* = 6;Student’s t-test was used for significance test and data were presented as the means± SEM) (PDF 33853 kb)

## Abbreviations

AUC: Area under curve
CI: Confidence interval
circRNAs: Circular RNAs
DE: Differentially expressed
DFS: Disease-free survival
EAC: Esophageal adenocarcinoma
EC: Esophageal cancer
ESCC: Esophageal squamous cell carcinoma
FBS: Fetal bovine serum
FFPE: Formalin fixed and paraffin embedded
FISH: Fluorescence in situ hybridization
GO: Gene oncology
HR: Hazard ratio
KEGG: Kyoto encyclopedia of genes and genomes
MREs: miRNA-responsive elements
NSCLC: Non-small cell lung cancer
OS: Overall survival
qRT-PCR: Quantitative real-time polymerase chain reaction
ROC: Receiver operating characteristic
SEM: Standard error of the mean

## References

[1] Thrift AP (2016). The epidemic of oesophageal carcinoma: where are we now?. Cancer Epidemiol.

[2] Torre LA, Bray F, Siegel RL, Ferlay J, Lortet-Tieulent J, Jemal A (2015). Global cancer statistics, 2012. CA Cancer J Clin.

[3] Siegel RL, Miller KD, Jemal A (2017). Cancer statistics, 2017. CA Cancer J Clin.

[4] Barrett SP, Salzman J (2016). Circular RNAs: analysis, expression and potential functions. Development.

[5] Li Y, Zheng Q, Bao C, Li S, Guo W, Zhao J, Chen D, Gu J, He X, Huang S (2015). Circular RNA is enriched and stable in exosomes: a promising biomarker for cancer diagnosis. Cell Res.

[6] Zhang M, Xin Y, Circular RNA (2018). A new frontier for cancer diagnosis and therapy. J Hematol Oncol.

[7] Zhu J, Ye J, Zhang L, Xia L, Hu H, Jiang H, Wan Z, Sheng F, Ma Y, Li W (2017). Differential expression of Circular RNAs in glioblastoma Multiforme and its correlation with prognosis. Transl Oncol.

[8] Yao JT, Zhao SH, Liu QP, Lv MQ, Zhou DX, Liao ZJ, Nan KJ (2017). Over-expression of CircRNA_100876 in non-small cell lung cancer and its prognostic value. Pathol Res Pract.

[9] Lu C, Shi X, Wang AY, Tao Y, Wang Z, Huang C, Qiao Y, Hu H, Liu L (2018). RNA-Seq profiling of circular RNAs in human laryngeal squamous cell carcinomas. Mol Cancer.

