# Correction to: Circular RNA profiling and its potential for esophageal squamous cell cancer diagnosis and prognosis

**DOI:** 10.1186/s12943-019-1050-y

**Published:** 2019-08-05

**Authors:** Liyuan Fan, Qiang Cao, Jia Liu, Junpeng Zhang, Baosheng Li

**Affiliations:** 1 Department of Radiation Oncology, Shandong Cancer Hospital Affiliated to Shandong University, Jinan, 250117 China; 2 School of Computer Science and Engineering, Southeast University, Nanjing, China; 3 Department of Clinical Laboratory, The Second Hospital of Shandong University, Jinan, China

**Correction to: Mol Cancer**

**https://doi.org/10.1186/s12943-018-0936-4**

After publication of the article [1], an error was noticed in the Funding section. The funding number provided was 2016YFC0105100 and should be corrected to 2016YFC0105106. We apologize for this error, which does not affect any of the interpretations or conclusions of the article.
