# Correction to: Circular RNA profiling and its potential for esophageal squamous cell cancer diagnosis and prognosis

**DOI:** 10.1186/s12943-020-01230-5

**Published:** 2020-07-02

**Authors:** Liyuan Fan, Qiang Cao, Jia Liu, Junpeng Zhang, Baosheng Li

**Affiliations:** 1 Department of Radiation Oncology, Shandong Cancer Hospital affiliated to Shandong University, Jinan, 250117 China; 2 School of Computer Science and Engineering, Southeast University, Nanjing, China; 3 Department of Clinical Laboratory, the Second Hospital of Shandong University, Jinan, China

**Correction to: Mol Cancer (2019) 18:16**

**https://doi.org/10.1186/s12943-018-0936-4**

Following the publication of [1], we recently found some errors in the manuscripts and figures. In text section “Hsa_circ_0001946 as a prognostic biomarker in both frozen and FFPE tissues” of Page 3, we wrote “longer” as “shorter” in the manuscripts. The corrected context is presented as below.

**Hsa_circ_0001946 as a prognostic biomarker in both frozen and FFPE tissues**

As Additional file 6: Table S2 showed, hsa_circ_0001946 was the only one associated with the recurrence rate of ESCC patients. As for disease-free survival (DFS) and overall survival (OS) prediction, patients in the high hsa_circ_0001946 group (according to the median level) had a much longer DFS and OS (Fig. 2a and Fig. 2e), whose hazard ratio (HR) and 95% confidence interval (CI) were 0.357(0.164–0.781) and 0.209(0.076–0.579) respectively.

Furthermore, we used FFPE tissues to verify this conclusion. The results showed that high hsa_circ_0001946 was associated with longer DFS and OS in K-M curves (Fig. 2b and Fig. 2f) while univariate Cox proportional hazard models showed that expression of hsa_circ_0001946 in FFPE tissues was an independent prognostic indicator of OS but not DFS. The multivariate Cox proportional hazard models shown that combination of these two group supports the result that hsa_circ_0001946 was a promising and independent prognostic biomarker for ESCC patients in both frozen and FFPE tissues.

In Figure 3E, the legend of tumor growth curve (left panel) was reversedly marked between Eca109-V and Eca109-N group. The modified plot with corrected legend were presented below.

**Figure 3E**

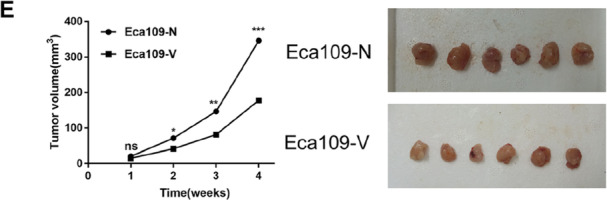



In Figure S7, the image of TE-1-V in migration assay (Figure S7I) and the image of wound-healing assay of K50-N (24h, median column Figure S7E) were arranged in wrong placed. These image errors were caused by repeatedly selecting, below is the corrected images.

**Figure S7E**

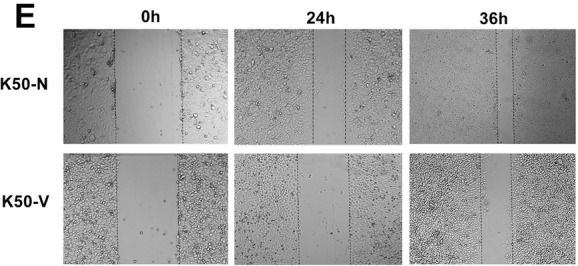



**Figure S7I**

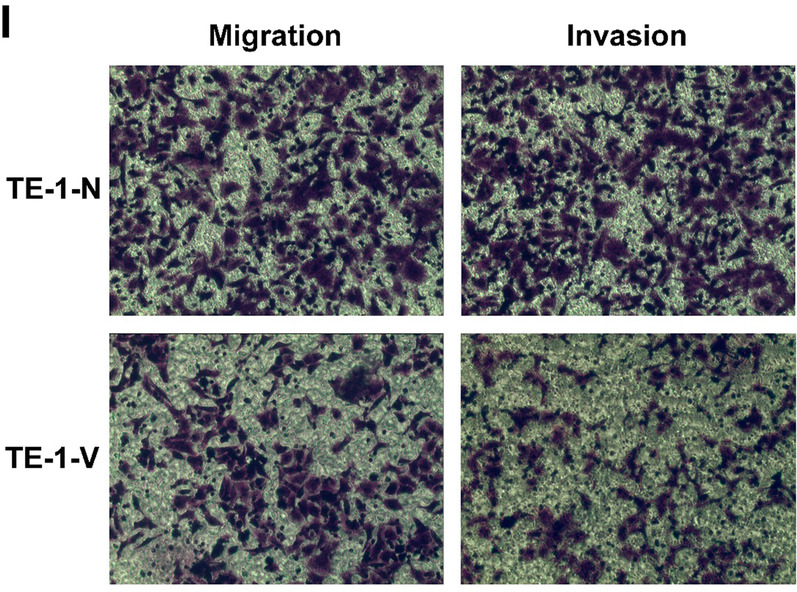



These errors were caused by our carelessness and misoperation in image combination. After carefully re-checked the manuscripts, raw data, and lab records. We assure that the correction of these will not alter the conclusion of the results. We sincerely apologize for the errors and any confusion it may have caused.
