# Supplementary figures and images for: Circular RNA profiling and its potential for esophageal squamous cell cancer diagnosis and prognosis
Source: Mol Cancer. 2019 Jan 23;18:16. doi: 10.1186/s12943-018-0936-4 (PMC6343327; doi:10.1186/s12943-018-0936-4)

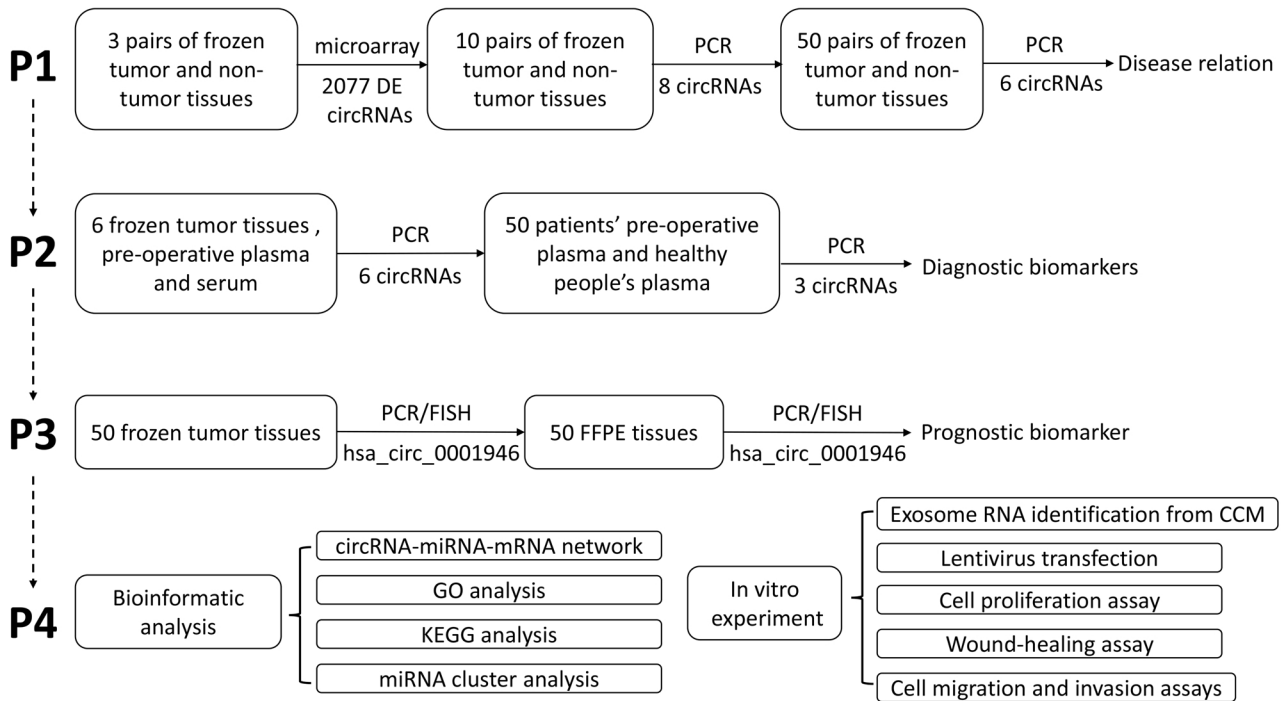

Supplement: Supplementary file 1 — Figure S1. Flowchart of our study. P1 to P4 represents four parts. DE: differentially expressed; PCR: Polymerase Chain Reaction; FISH: Fluorescence In Situ Hybridization; FFPE: formalin fixed paraffin-embedded; GO: Gene Ontology; KEGG: Kyoto Encyclopedia of Genes and Genomes. (PDF 2574 kb) [file 12943_2018_936_MOESM1_ESM.pdf]

A

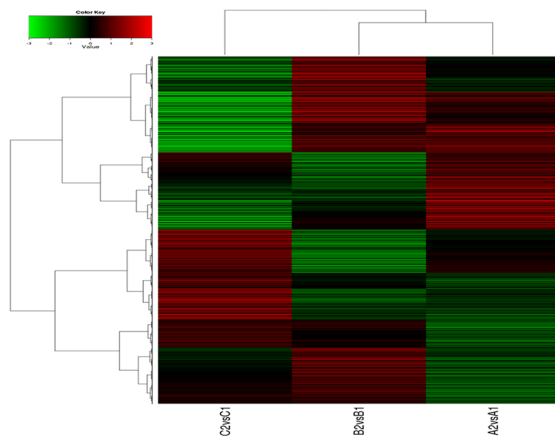

B

Log-Log Scatter Plot

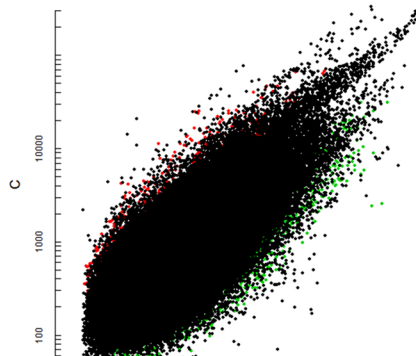

C

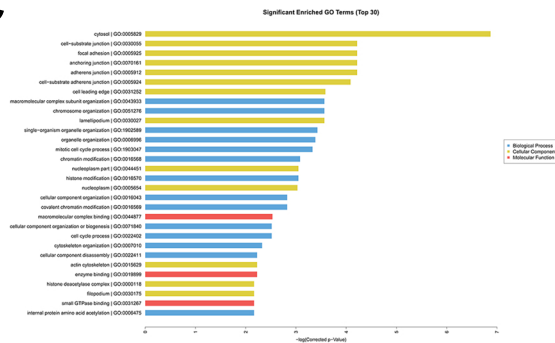

D

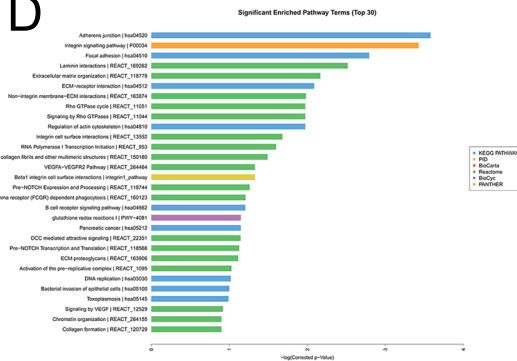

Supplement: Supplementary file 2 — Figure S2. Screening of differentially expressed circRNAs by circRNA microarray and functional annotation of their host genes. (A) Hierarchical clustering results of circRNAs expression profiles among 3 pairs of ESCC tumor tissues and non-tumor tissues. Red represented relatively high expression while green represented relatively low expression. A1~C1 were tumor tissues while A2~C2 were non-tumor tissues. (B) Red dots in the scatter-Plot indicated high expressed circRNAs while green dots here indicated low expressed circRNAs. (C) Go analysis of host genes was performed to obtain three categories (cellular component, molecular function, and biological process). (D) The top 30 signaling pathways potentially involved in the circRNA-mediated regulatory network in ESCC by KEGG analysis of host genes. (PDF 3924 kb) [file 12943_2018_936_MOESM2_ESM.pdf]

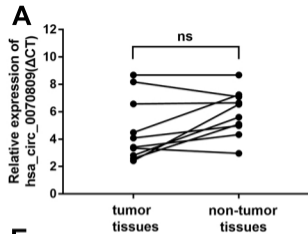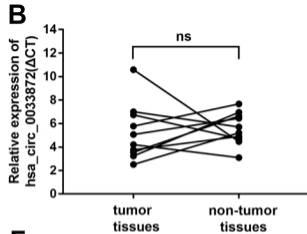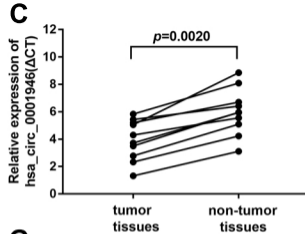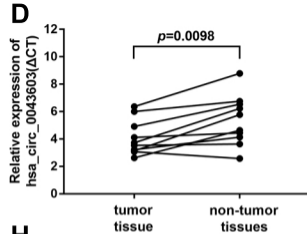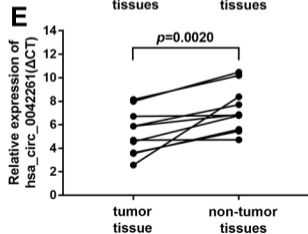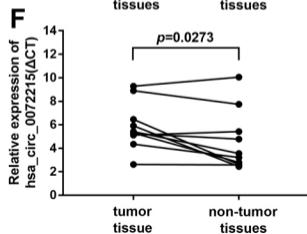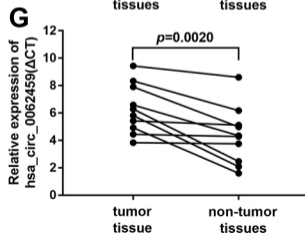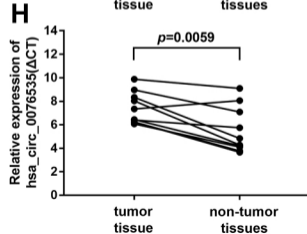

Supplement: Supplementary file 4 — Figure S3. Confirmation of differentially expressed circRNAs by qRT-PCR in frozen tumor and non-tumor tissues. (A)~(H)The expression levels of 8 circRNAs(hsa_circ_0062459, hsa_circ_0076535, hsa_circ_0072215, hsa_circ_0033872, hsa_circ_0042261, hsa_circ_0070809, hsa_circ_0001946 and hsa_circ_0043603) were detected by qRT-PCR in 10 pairs of frozen tissues. Mann-Whitney test was used for the significance test. (PDF 1728 kb) [file 12943_2018_936_MOESM4_ESM.pdf]

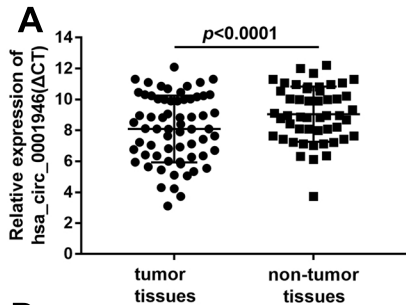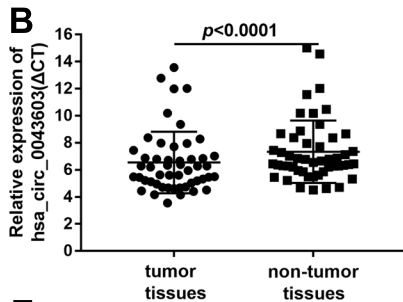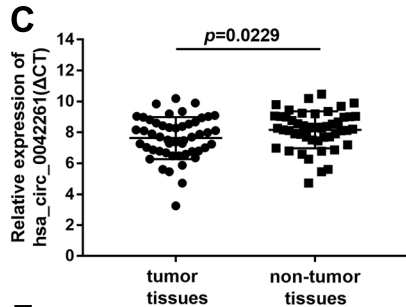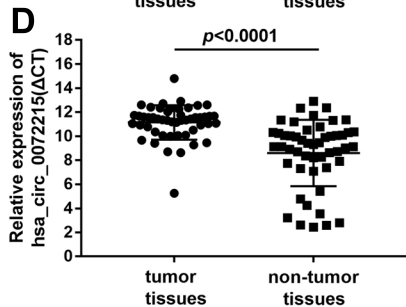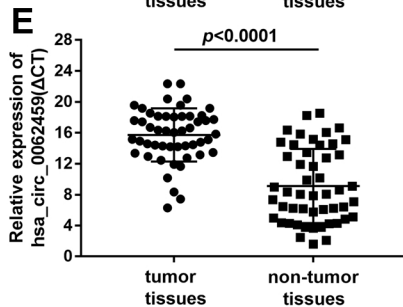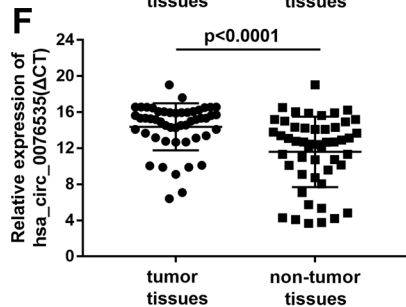

Supplement: Supplementary file 5 — Figure S4. Confirmation of differentially expressed circRNAs by qRT-PCR in frozen tumor and non-tumor tissues. (A)~(F) The expression levels of 6 circRNAs(hsa_circ_0062459, hsa_circ_0076535, hsa_circ_0072215, hsa_circ_0042261, hsa_circ_0001946, and hsa_circ_0043603) were detected by qRT-PCR in 50 pairs of frozen tissues. Student’s t-test was used for significance test. (PDF 2114 kb) [file 12943_2018_936_MOESM5_ESM.pdf]

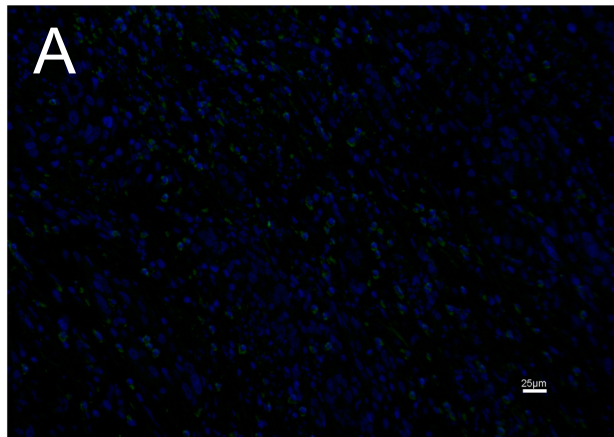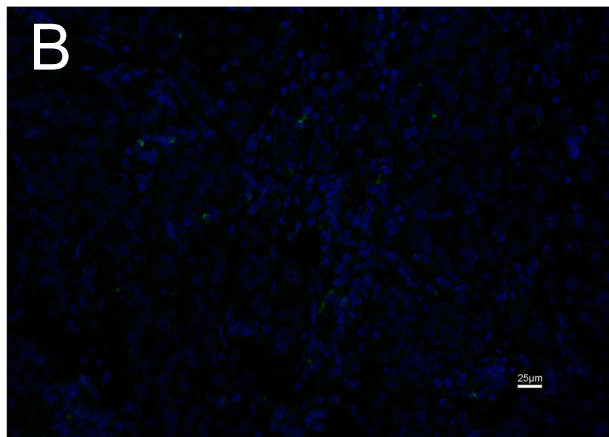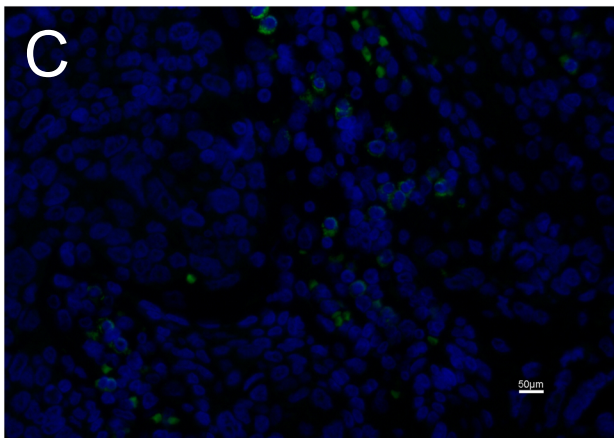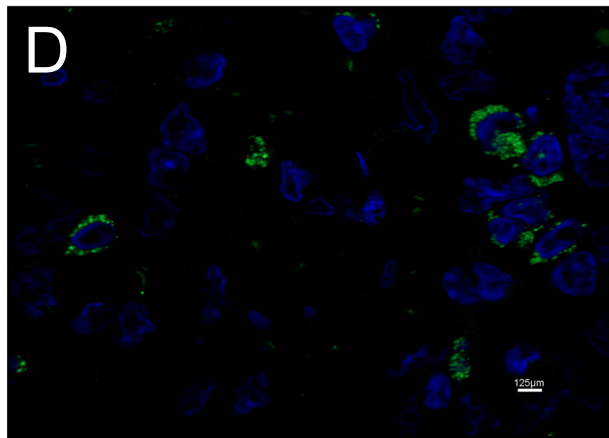

Supplement: Supplementary file 9 — Figure S5. Semi-quantitation and location of hsa_circ_0001946 by FISH in FFPE tissues. (A~B) High and low expression levels of hsa_circ_0001946 in samples by Immunofluorescence Accumulation Optical Density (IOD) analysis under 200X condition. (C) Expression pattern of hsa_circ_0001946 under 400X condition. (D) This image was recorded on a wide-field fluorescence microscope via a 63× oil objective. It represented the localization of hsa_circ_0001946 in cells. The blue color was stained nuclei by DAPI, and the green color was stained hsa_circ_0001946 in the cytoplasm. (PDF 32764 kb) [file 12943_2018_936_MOESM9_ESM.pdf]

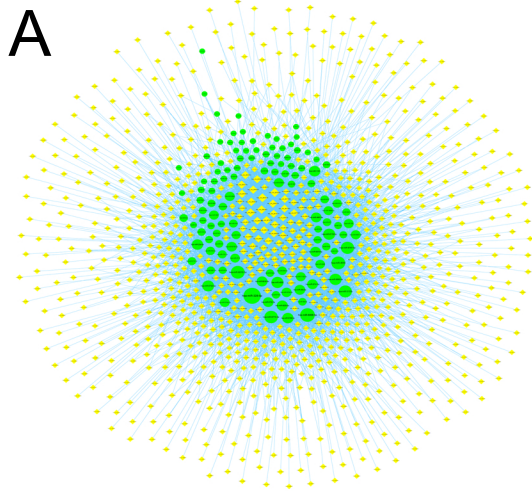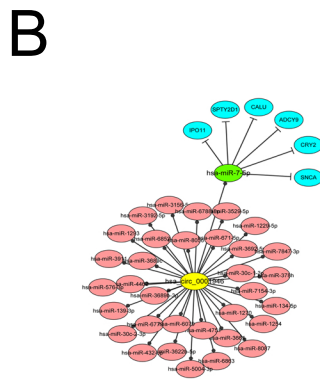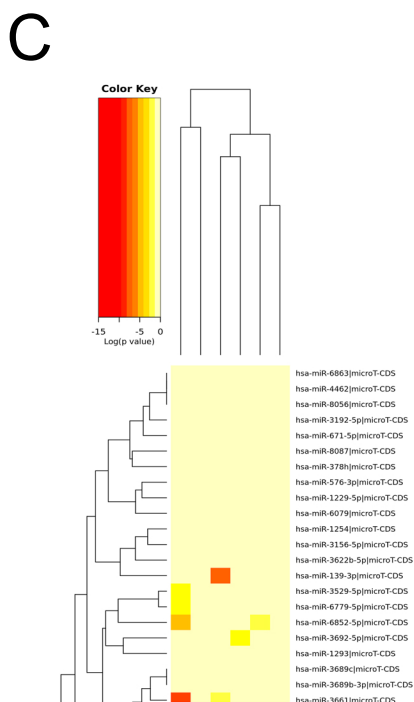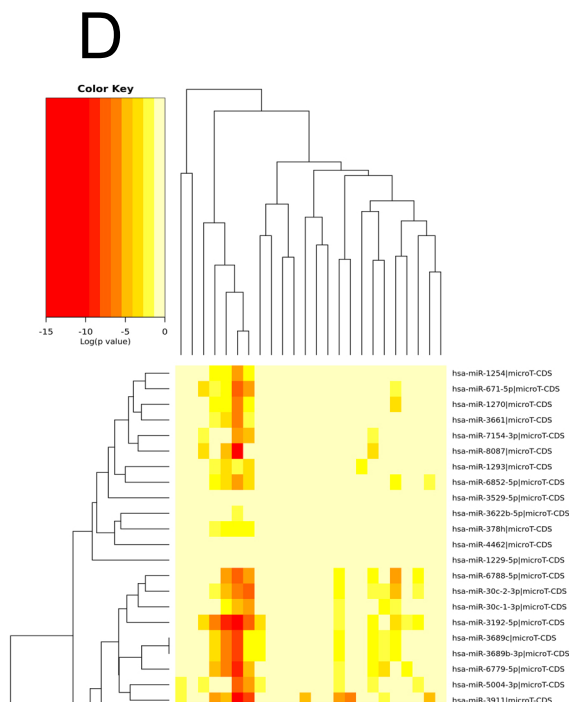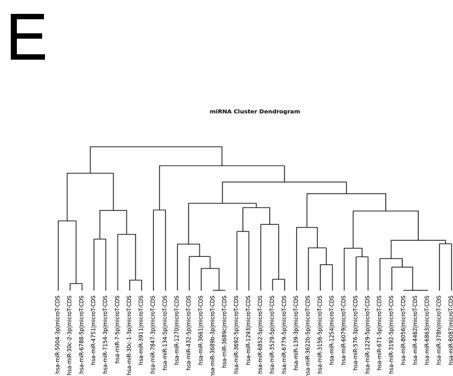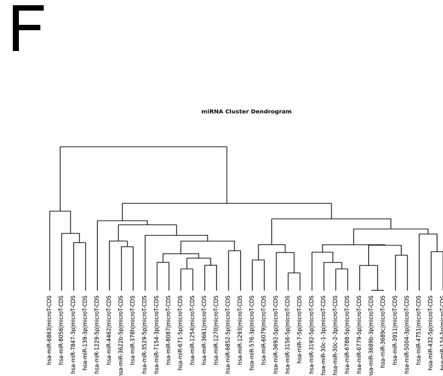

Supplement: Supplementary file 11 — Figure S6. Prediction an annotation of hsa_circ_0001946 targeted miRNA-mRNA network. (A) The coexpression network of circRNAs and miRNAs obtained by microarray tested in the same three pairs of ESCC tumor tissues and non-tumor tissues. Green ones represent miRNAs while yellow ones represent circRNAs. (B) Hsa_circ_0001946 targeted miRNA-mRNA network combined with the microarray results and predicted by several algorithms. Yellow one represents algorithms hsa_circ_0001946. Pink and green ones represent targeted miRNAs of hsa_circ_0001946. Blue ones represent targeted mRNAs of hsa-miR-7-5P. (C~D) The GO and KEGG analysis of the targeted miRNAs of hsa_circ_0001946. (E~F) The miRNA cluster analysis of the targeted miRNAs of hsa_circ_0001946. (PDF 9920 kb) [file 12943_2018_936_MOESM11_ESM.pdf]

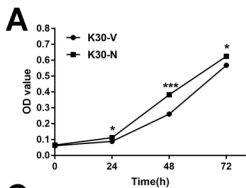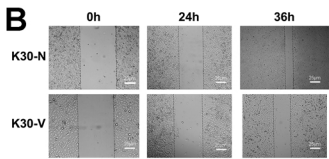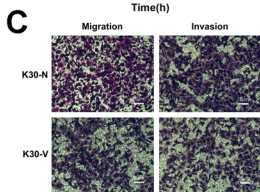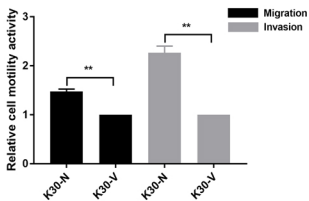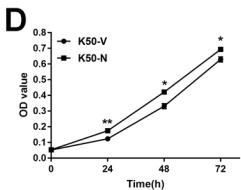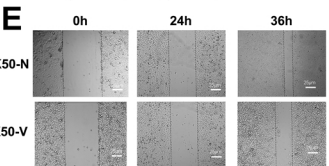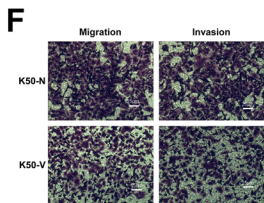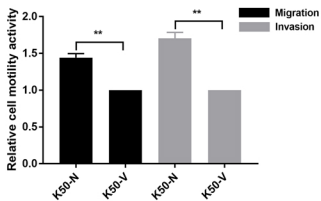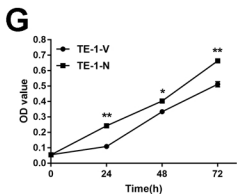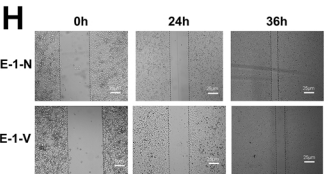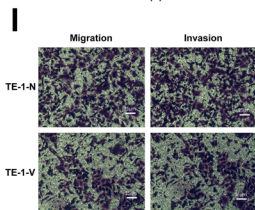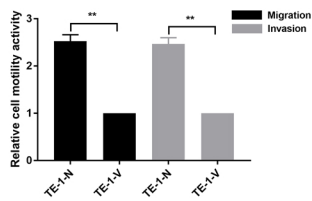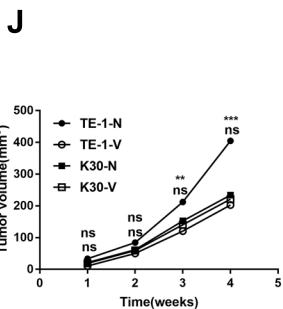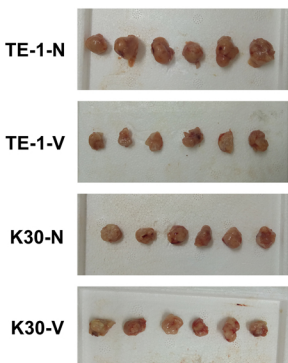

Supplement: Supplementary file 12 — Figure S7. Hsa_circ_0001946 overexpression affects the proliferation, migration, and invasion of K30, K50, and TE-1. (A)(D)(G) MTT method showed that hsa_circ_0001946 overexpression inhibited K30, K50, and TE-1 proliferation after 24 h. (B)(E)(H) Wound-healing assay indicated that hsa_circ_0001946 overexpression decreased K30, K50 and TE-1 migration after 24 h. (C)(F)(I) Transwell assays showed that hsa_circ_0001946 overexpression decreased K30, K50 and TE-1 migration and invasion after 48 h incubation. (n = 3; Student’s t-test was used for significance test and data were presented as the means± SEM.) (J)Subcutaneous xenografts excised from nude mice. (n = 6;Student’s t-test was used for significance test and data were presented as the means± SEM) (PDF 33853 kb) [file 12943_2018_936_MOESM12_ESM.pdf]
