# Supplementary material for: Circular RNA profiling and its potential for esophageal squamous cell cancer diagnosis and prognosis
Source: Mol Cancer. 2019 Jan 23;18:16. doi: 10.1186/s12943-018-0936-4 (PMC6343327; doi:10.1186/s12943-018-0936-4)
Supplement: Supplementary file 3 — Table S1. Selected DE circRNAs by microarray and validation by qRT-PCR. *number of MRE targeted by the related circRNA; DE: differentially expressed; FC: fold change; MRE: miRNA response elements (DOCX 17 kb) [file 12943_2018_936_MOESM3_ESM.docx]

**Table S1.** Selected DE circRNAs by microarray and validation by qRT-PCR. *number of MRE targeted by the related circRNA; DE: differentially expressed; FC: fold change; MRE: miRNA response elements

| circRNA | *p* value | FC | Regulation | Gene  Symbol | Annotation | MRE | qRT-PCR |
| --- | --- | --- | --- | --- | --- | --- | --- |
| hsa_circ_0062459 | 0.0117 | 4.47 | Up | None | INTERGENIC | hsa-let-7a-2-3p;  hsa-let-7a-5p etc.(1812*) | Up |
| hsa_circ_0076535 | 0.0411 | 16.79 | Up | POLR1C | ANNOTATED, CDS, coding, OVCODE, OVERLAPTX, OVEXON, UTR3, UTR5 | hsa-miR-125a-3p;  hsa-miR-1915-3p etc.(16) | Up |
| hsa_circ_0072215 | 0.0398 | 16.39 | Up | C5orf42 | ANNOTATED, CDS, coding, INTERNAL, OVCODE, OVEXON | hsa-miR-3714.(1) | Up |
| hsa_circ_0033872 | 0.0012 | 3.82 | Up | ADAM6 | ALT_ACCEPTOR, ALT_DONOR, downstream end, ncRNA, et.al | hsa-let-7a-2-3p;  hsa-let-7a-5p etc.(2414) | - |
| hsa_circ_0042261 | 0.0445 | 6.20 | Down | TOM1L2 | ANNOTATED, CDS, coding, INTERNAL, OVCODE, OVEXON | hsa-miR-1227-5p;  hsa-miR-1231.(29) | Down |
| hsa_circ_0070809 | 0.0463 | 11.89 | Down | PDE5A | ANNOTATED, CDS, coding, INTERNAL, OVCODE, OVERLAPTX, OVEXON | hsa-miR-29a-5p;  hsa-miR-635 etc.(4) | - |
| hsa_circ_0001946 | 0.0172 | 40.21 | Down | CDR1 | ANTISENSE, CDS, coding, downstream end, upstream start, UTR3, UTR5 | hsa-miR-1229-5p;  hsa-miR-1254 etc.(34) | Down |
| hsa_circ_0043603 | 0.0043 | 3.25 | Down | KRT19 | ALT_ACCEPTOR, CDS, coding, OVCODE, UTR5, OVERLAPTX, UTR3, et.al | hsa-let-7a-2-3p;  hsa-let-7a-5p etc.(2198) | Down |
