# Supplementary material for: Circular RNA profiling and its potential for esophageal squamous cell cancer diagnosis and prognosis
Source: Mol Cancer. 2019 Jan 23;18:16. doi: 10.1186/s12943-018-0936-4 (PMC6343327; doi:10.1186/s12943-018-0936-4)
Supplement: Supplementary file 6 — Table S2. Relationships of circRNAs expression levels in ESCC frozen tumor tissues with clinicopathological characteristics by qRT-PCR. (DOCX 21 kb) [file 12943_2018_936_MOESM6_ESM.docx]

**Table 2.** Relationships of circRNAs expression levels in ESCC frozen tumor tissues with clinicopathological characteristics by qRT-PCR.

| Characteristics | No. of patients | hsa_circ_0062459 | | hsa_circ_0076535 | | hsa_circ_0072215 | | hsa_circ_0042261 | | hsa_circ_0001946 | | hsa_circ_0043603 | |
| --- | --- | --- | --- | --- | --- | --- | --- | --- | --- | --- | --- | --- | --- |
|  | (%) | Median | *p* | Median | *p* | Median | *p* | Median | *p* | Median | *p* | Median | *p* |
| Age(year)  ≤ 60  >60 | 25 (50)  25 (50) | 15.82  16.34 | 0.7766 | 15.18  15.32 | 0.6270 | 11.44  11.34 | 0.6685 | 7.69  7.71 | 0.7399 | 7.43  7.23 | 0.6757 | 5.50  6.29 | 0.5096 |
| Gender  Female  Male | 17 (34)  33 (66) | 16.25  15.15 | 0.4804 | 15.33  14.93 | 0.3264 | 11.31  11.56 | 0.3849 | 7.71  7.69 | 0.9800 | 6.35  8.51 | **0.0059** | 6.72  5.96 | 0.3961 |
| Diameter(cm)  ≤ 4  > 4 | 29 (58)  21 (42) | 16.34  15.82 | 0.6157 | 15.18  15.32 | 0.2015 | 11.36  11.44 | 0.8495 | 7.88  7.69 | 0.4266 | 8.12  6.35 | **0.0062** | 6.78  5.50 | 0.0743 |
| Gross type  Ulcerative type  Other types | 29 (58)  21 (42) | 16.25  15.15 | 0.5041 | 15.18  15.26 | 0.7515 | 11.56  11.36 | 0.3166 | 7.88  7.37 | 0.5105 | 7.43  7.23 | 0.8494 | 6.66  5.61 | 0.1375 |
| Differentiation  Well&Moderate  Poor | 20 (40)  30 (60) | 14.49  16.25 | 0.1982 | 14.54  15.33 | 0.2052 | 11.26  11.40 | 0.4641 | 7.42  7.81 | 0.4642 | 7.77  7.02 | 0.5394 | 5.56  6.28 | 0.9960 |
| TNM stage  0~ II  III~ IV | 21 (42)  29 (58) | 14.77  17.59 | **0.0121** | 15.26  15.18 | 0.7515 | 11.07  11.56 | **0.0436** | 7.37  7.88 | 0.5105 | 8.51  6.76 | **0.0219** | 6.29  5.45 | **0.0118** |
| Invasion  Tis&T2  T3&T4 | 21 (42)  29 (58) | 16.69  15.11 | 0.3263 | 14.93  15.33 | 0.1271 | 10.78  11.56 | **0.0033** | 8.11  7.3 | 0.5887 | 8.82  6.70 | **0.0140** | 6.34  5.55 | 0.0695 |
| Lymphatic metastasis  N0  N1~N3 | 26 (52)  24 (48) | 14.43  17.06 | **0.0181** | 14.44  15.48 | **0.0212** | 11.35  11.56 | 0.2034 | 7.71  7.70 | 0.8138 | 8.69  6.35 | **0.0040** | 6.28  5.42 | 0.1505 |
| Distal metastasis  M0  M1 | 31 (62)  19 (38) | 16.25  16.04 | 0.7327 | 15.26  15.18 | 0.6522 | 11.44  11.36 | 0.7177 | 7.47  8.16 | 0.5228 | 7.91  6.70 | 0.1049 | 5.61  6.66 | 0.2193 |
| CEA  Positive  Negative | 21 (42)  29 (58) | 17.06  14.86 | 0.0581 | 15.33  14.18 | **0.0126** | 11.56  10.98 | **0.0050** | 7.70  7.67 | 0.5447 | 6.56  8.91 | **0.0036** | 5.55  6.32 | **0.0311** |
| Cyfra21-1  Positive  Negative | 30 (60)  20 (40) | 16.47  14.94 | **0.0381** | 15.48  14.20 | **0.0177** | 11.56  10.91 | **0.0486** | 7.42  8.11 | 0.1751 | 6.55  9.56 | **0.0001** | 5.62  6.31 | **0.0311** |
| Recurrence  Yes  No | 21 (42)  29 (58) | 14.57  16.63 | 0.0554 | 15.32  14.93 | 0.8038 | 11.44  11.36 | 0.7003 | 7.9  7.69 | 0.9030 | 6.35  8.12 | **0.0077** | 5.61  6.28 | 0.7369 |
