# Supplementary material for: Circular RNA profiling and its potential for esophageal squamous cell cancer diagnosis and prognosis
Source: Mol Cancer. 2019 Jan 23;18:16. doi: 10.1186/s12943-018-0936-4 (PMC6343327; doi:10.1186/s12943-018-0936-4)
Supplement: Supplementary file 7 — Table S3. Average expression of 6 circRNAs in 6 ESCC patients’ samples and 6 healthy people’s samples. *It means hsa_circ_0042261 was detected in one samples among total 6 samples (DOCX 14 kb) [file 12943_2018_936_MOESM7_ESM.docx]

**Supplementary Table S3.** Average expression of 6 circRNAs in 6 ESCC patients’ samples and 6 healthy people’s samples. *It means hsa_circ_0042261 was detected in one samples among total 6 samples

|  | Tumor tissues | Non-tumor tissues | Patients’ plasma | Patients’ serum | Healthy people’s plasma | Healthy people’s serum |
| --- | --- | --- | --- | --- | --- | --- |
| hsa_circ_0001946 | 9.114 | 7.727 | -0.658 | -0.062 | 0.748 | 0.622 |
| hsa_circ_0042261 | 7.111 | 8.215 | 1/6* | 1/6 | 2/6 | 2/6 |
| hsa_circ_0043603 | 5.235 | 6.401 | -1.348 | -2.158 | 4.963 | 1.25 |
| hsa_circ_0062459 | 17.786 | 14.087 | 7.911 | 2.988 | 1.847 | 1.694 |
| hsa_circ_0072215 | 11.406 | 9.253 | 1/6 | 0/6 | 3/6 | 2/6 |
| hsa_circ_0076535 | 15.972 | 14.565 | 1/6 | 0/6 | 1/6 | 0/6 |
