# Supplementary material for: Circular RNA profiling and its potential for esophageal squamous cell cancer diagnosis and prognosis
Source: Mol Cancer. 2019 Jan 23;18:16. doi: 10.1186/s12943-018-0936-4 (PMC6343327; doi:10.1186/s12943-018-0936-4)
Supplement: Supplementary file 8 — Table S4. Relationships of circRNAs expression levels in plasma of ESCC patients with clinicopathological characteristics by qRT-PCR. (DOCX 18 kb) [file 12943_2018_936_MOESM8_ESM.docx]

**Table S4.** Relationships of circRNAs expression levels in plasma of ESCC patients with clinicopathological characteristics by qRT-PCR.

| Characteristics | No. of patients | hsa_circ_0062459 | | hsa_circ_0001946 | | hsa_circ_0043603 | | Signature | |
| --- | --- | --- | --- | --- | --- | --- | --- | --- | --- |
|  | (%) | Median | *p* | Median | *p* | Median | *p* | Median | *p* |
| Age(year)  ≤ 60  >60 | 24 (48)  26 (52) | 8.64  9.08 | 0.6894 | -0.23  -0.30 | 0.6542 | 3.79  3.89 | 0.9655 | 1.64  2.25 | 0.4551 |
| Gender  Female  Male | 24 (48)  26 (52) | 9.36  8.07 | **0.0442** | -0.17  -0.40 | 0.1559 | 4.94  3.52 | 0.1293 | 1.03  2.27 | **0.0213** |
| Gross type  Ulcerative type  Other types | 26 (52)  24 (48) | 9.86  6.83 | **0.0088** | -0.26  -0.33 | 0.9348 | 3.65  4.04 | 0.9731 | 1.77  2.01 | 0.7987 |
| Differentiation  Well & Moderate  Poor | 24 (48)  26 (52) | 9.62  8.64 | 0.2859 | -0.41  -0.21 | 0.1268 | 3.49  4.94 | **0.0290** | 2.27  1.04 | **0.0284** |
| TNM stage  0~ II  III~ IV | 21 (42)  29 (58) | 9.37  8.65 | 0.8341 | -0.08  -0.42 | 0.0480 | 4.04  3.75 | 0.1321 | 1.31  2.14 | **0.0250** |
| Invasion  Tis~T2  T3~T4 | 23 (46)  27 (54) | 8.65  8.79 | 0.6746 | -0.28  -0.26 | 0.9346 | 4.93  3.75 | 0.4958 | 1.65  1.95 | 0.7760 |
| Lymphatic metastasis  N0  N1~N3 | 23 (46)  27 (54) | 8.79  8.22 | 0.7030 | -0.27  -0.28 | 0.8735 | 3.51  4.47 | 0.4776 | 2.11  1.63 | 0.6397 |
| Distal metastasis  M0  M1 | 28 (56)  22 (44) | 9.08  8.64 | 0.6108 | -0.27  -0.30 | 0.7308 | 3.53  4.94 | 0.1369 | 2.03  1.49 | 0.5191 |
| CEA  Positive  Negative | 28 (56)  22 (44) | 8.71  9.26 | 0.4177 | -0.43  -0.16 | **0.0360** | 3.54  5.00 | **0.0438** | 2.26  0.86 | **0.0052** |
| Cyfra21-1  Positive  Negative | 29 (58)  21 (42) | 9.37  8.65 | 0.5103 | -0.42  -0.17 | **0.0263** | 3.51  5.05 | 0.0191 | 2.14  0.74 | **0.0022** |
