# Supplementary material for: Circular RNA profiling and its potential for esophageal squamous cell cancer diagnosis and prognosis
Source: Mol Cancer. 2019 Jan 23;18:16. doi: 10.1186/s12943-018-0936-4 (PMC6343327; doi:10.1186/s12943-018-0936-4)
Supplement: Supplementary file 10 — Table S5. Univariate and multivariate Cox regression analyze hsa_circ_0001946 for overall survival (OS) and disease-free survival (DFS) of patients in frozen tumor tissue, FFPE tissue and plasma of ESCC patients. (DOCX 17 kb) [file 12943_2018_936_MOESM10_ESM.docx]

**Table S5.** Univariate and multivariate Cox regression analyze hsa_circ_0001946 for overall survival (OS) and disease-free survival (DFS) of patients in frozen tumor tissue, FFPE tissue and plasma of ESCC patients.

| Variables | HR (OS) | 95% (OS) | *p* (OS) | HR (DFS) | 95% (DFS) | *p*(DFS) |
| --- | --- | --- | --- | --- | --- | --- |
| Univariate analysis |  |  |  |  |  |  |
| Gender(Male vs. Female) | 0.844 | 0.436-1.636 | 0.616 | 0.805 | 0.457-1.419 | 0.454 |
| Age(>60 vs. ≤ 60) | 1.044 | 0.559-1.949 | 0.893 | 1.134 | 0.664-1.935 | 0.646 |
| Differentiation(Well + Moderately vs. Poorly + Undifferentiated) | 0.906 | 0.517-1.795 | 0.906 | 1.066 | 0.624-1.818 | 0.816 |
| Tumor size( ≤ 4 cm vs. > 4 cm) | 0.834 | 0.443-1.568 | 0.573 | 1.026 | 0.600-1.757 | 0.924 |
| Gross type(ulcerative type vs. other types) | 1.087 | 0.581-2.032 | 0.794 | 1.040 | 0.603-1.791 | 0.889 |
| TNM stage(I+II vs. III+IV) | 0.464 | 0.240-0.898 | 0.023 | 0.555 | 0.319-0.967 | 0.037 |
| Invasion depth (Tis ~T2 vs.T3 ~ T4) | 0.465 | 0.238-0.907 | 0.025 | 0.686 | 0.396-1.189 | 0.180 |
| Lymphatic metastasis (N0 vs. N1~ N3) | 0.452 | 0.236-0.868 | 0.017 | 0.556 | 0.323-0.955 | 0.034 |
| Distant metastasis (No vs. Yes) | 0.453 | 0.240-0.856 | 0.015 | 0.597 | 0.345-1.030 | 0.064 |
| CEA (Negative vs. Positive) | 0.545 | 0.281-1.057 | 0.073 | 0.586 | 0.338-1.018 | 0.058 |
| Cyfra21-1 (Negative vs. Positive) | 0.421 | 0.215-0.824 | 0.012 | 0.520 | 0.299-0.905 | 0.021 |
| Expression of hsa_circ_0001946 in frozen tissue by qRT-PCR (High vs. Low) | 0.209 | 0.076-0.579 | 0.003 | 0.357 | 0.164-0.781 | 0.010 |
| Expression of hsa_circ_0001946 in FFPE tissues by qRT-PCR (High vs. Low) | 0.294 | 0.106-0.818 | 0.019 | 0.486 | 0.212-1.117 | 0.089 |
| Expression of hsa_circ_0001946 by qRT-PCR (High vs. Low) | 0.243 | 0.118-0.504 | 0.000 | 0.418 | 0.235-0.742 | 0.003 |
| Expression of hsa_circ_0001946 in FFPE by FISH (High vs. Low) | 0.278 | 0.137-0.563 | 0.000 | 0.421 | 0.235-0.754 | 0.004 |
| Multivariate analysis |  |  |  |  |  |  |
| TNM stage(I+II vs. III+IV) | 0.816 | 0.379-1.755 | 0.043 | 0.795 | 0.413-1.531 | 0.047 |
| Invasion depth (Tis ~T2 vs.T3 ~ T4) | 0.682 | 0.335-1.387 | 0.291 | 0.865 | 0.483-1.548 | 0.625 |
| Lymphatic metastasis (N0 vs. N1~ N3) | 0.770 | 0.360-1.647 | 0.500 | 0.840 | 0.438-1.609 | 0.599 |
| Distant metastasis (No vs. Yes) | 0.776 | 0.392-1.536 | 0.467 | 0.813 | 0.454-1.456 | 0.486 |
| Cyfra21-1 (Negative vs. Positive) | 0.722 | 0.347-1.502 | 0.384 | 0.681 | 0.376-1.231 | 0.203 |
| Expression of hsa_circ_0001946 by qRT-PCR (High vs. Low) | 0.385 | 0.164-0.904 | 0.028 | 0.575 | 0.300-1.100 | 0.049 |
| Expression of hsa_circ_0001946 by FISH (High vs. Low) | 0.401 | 0.162-0.995 | 0.049 | 0.414 | 0.190-0.904 | 0.027 |
